# Changes in body weight and blood pressure among women using Depo-Provera injection in Northwest Ethiopia

**DOI:** 10.1186/s13104-019-4555-y

**Published:** 2019-08-15

**Authors:** Muluken Fekadie Zerihun, Tabarak Malik, Yohannes Mulu Ferede, Tesfahun Bekele, Yigizie Yeshaw

**Affiliations:** 1 Department of Biochemistry, School of Medicine, College of Medicine and Health Sciences, University of Gondar, Gondar, Ethiopia; 2 Nursing Department, College of Medicine and Health Sciences, University of Gondar, Gondar, Ethiopia; 3 Department of Physiology, School of Medicine, College of Medicine and Health Sciences, University of Gondar, Gondar, Ethiopia

**Keywords:** Body weight, Depo Provera, Blood pressure, Ethiopia

## Abstract

**Objectives:**

Depo-Provera is an injectable contraceptive method containing medroxyprogesterone acetate. It has some adverse effects like changes in menstrual pattern, loss in bone mineral density and risk of weight gain. Therefore, this study is aimed at to investigate the effects of Depo-Provera on body weight and blood pressure among Ethiopian women. Institution based cross-sectional study design was conducted from January 2017 to April 2017. Data were analyzed using SPSS version 21 software. Paired *t* test, independent t-test and ANOVA were used to evaluate the presence of mean difference and relationship between changes in variables and duration of use of Depo-Provera. P-value ≤ 0.05 were considered as statistically significant.

**Results:**

The mean weight and body mass index (BMI) of Depo-Provera users were increased significantly (p = 0.02 for mean body weight and p = 0.019, for body mass index). There was no significant difference in mean arterial blood pressure (MAP) of Depo-Provera users compared to controls or their respective pretreatment value (p-value = 0.85 for Depo-Provera users and 0.67 for non-users). The finding of this study revealed that there is an increased weight gain and BMI among Depo-Provera users compared to non-users, which really requires attention of health professionals and other stake holders.

## Introduction

Worldwide average fertility rate is 1.7 in developed countries compared to 4.6 children per woman in least developed countries [1]. In Ethiopia average total fertility rate is 4.1, which is among the highest in the world [2, 3]. Increased use of family planning methods help to bring these figures down to low levels in less developed countries like Ethiopia.

In Ethiopia, the family planning service is provided by both governmental and NGO health facilities, including hospitals, clinics, health centers, and health stations [4]. Among the different methods of family planning, injectable hormonal contraceptives; Depo-Provera (Depo-medroxyprogesterone acetate) are the most popular contraceptive methods in Sub-Saharan Africa including Ethiopia, contributing to about 40% of the total method mix in the region [5]. Depo-Provera is among a highly effective, convenient non-daily injectable hormonal contraceptive option with a very low failure rate that has been available worldwide for many years. The recommended dose of this contraceptive is 150 mg, administered by deep intramuscular (IM) injection at the interval of 3 months using strict aseptic technique in the gluteal or deltoid muscle [6, 7]. Side effects of this contraceptive method include: changes in menstrual patterns, loss of bone mineral density and risk of weight gain.

Prior studies have demonstrated the association between Depo-Provera with both weight gain [8, 9] and blood pressure [10]. On the other hand, another study reported that Depo-Provera injections did not cause a significant increase in body weight and blood pressure [6]. Since the previous studies performed among different ethnic groups showed diverse results and available data on this topic which are mostly from developed countries, this study was aimed to investigate the effects of Depo-Provera on body weight and blood pressure among Ethiopian women.

## Main text

### Methods

#### Study area and period

This study was conducted at Azezo Health Center, Gondar, Northwest Ethiopia. It serves for a total population of more than 25,000 individuals per year. The study was conducted from January 2017 to April 2017.

Study design: Institution-based comparative cross-sectional study design was employed.

#### Source and study population

All Depo-Provera users between 18 and 45 years of ages and the age matched non-Depo Provera users were the source population. Depo-Provera users between 18 and 45 years of age and age matched non-Depo Provera users who were present during the study period were the study population.

#### Inclusion and exclusion criteria

Women with chronic diseases like Diabetes mellitus and Hypertension and those who took medications known to affect body weight and blood pressure were excluded.

#### Sample size determination

The sample size for this study was determined by double-population formula: assuming 95% confidence interval, 80% power and by using the highest standard deviation. Systematic random sampling technique was used to select 50 healthy women who had been using Depo-Provera for at least 6 months and convenient sampling technique was used to recruit another 50 healthy age-matched (± 2 years) controls who visited the health centers accompanying the patients during the study period.

#### Variables of the study

Dependent variable: Body weight, body mass index and blood pressure.

Independent variables: Age, duration of use of Depo Provera.

#### Data collection instrument and procedure

Structured pretested interviewer administered questionnaire was used to collect data. The questionnaire was prepared in English first and translated to Amharic and then, retranslated back to English by another person to check its consistency. Blood pressure was measured using recently calibrated standardized sphygmomanometer. The height measurement was also taken using stadiometer on flooring that is not carpeted and against a flat surface such as a wall with no molding and by removing the shoes, bulky clothing, hair ornaments, and unbraid hair that interferes with the measurement. The weight (Kg) of study participants was taken using a mechanical scale or balance.

The Mean Arterial Blood Pressure (MAP) was calculated by using the formula, MAP = Diastolic Blood Pressure (DBP) + 1/3(Systolic Blood Pressure-DBP). Body Mass Index (BMI) was computed by dividing weight (Kg) to the square of height in meter. The change in body weight, BMI and MAP of Depo-Provera users were calculated as the difference between initial body weight, BMI and MAP on the first day of injection, recorded by the health care providers and the final weight, BMI and MAP taken at their visit during study period.

#### Data quality control

Training was given for data collectors and pretest was also done. During data collection, data collectors were closely supervised by the supervisor and principal investigator on daily basis. Then after appropriate data clean up and correction was done just before analysis.

#### Data processing and analysis

Data were entered into Epi Data version 3.1 and exported to Statistical package for Social Sciences (SPSS) version 21 for analysis. Descriptive statistics such as mean, standard deviation (SD) and range were calculated. Paired t-test was used to evaluate change in body weight, BMI and blood pressure of Depo-Provera users. Independent t-test was also used to compare the results of blood pressure of Depo-Provera users and their age-matched control group. One-way ANOVA was used to identify the variation of variables in relation to the duration of use of Depo-Provera. Pearson’s correlation-test was used to evaluate the association between the variables. All values were quoted as the mean ± SD, p-values of ≤ 0.05 were considered to be statistically significant.

### Results

#### Description of study participants

A total of 100 study participants (50 Depo-Provera users and 50 controls) were included in the study. The mean age of Depo-Provera users was 27.54 ± 5.63 years and 27.44 ± 5.02 years for control group. The mean BMI (Kg/m^2^) was 22.05 ± 3.10 for Depo-Provera users and 21.55 ± 3.05 for that of controls.

#### Magnitude and determinants of change in body weight and body mass index

This study indicates that Depo-Provera caused a significant weight gain. Weight gain varies from 1 to 14 kg. Excessive weight gain (≥ 10%) was observed in 9 (18%) of Depo-Provera users. Moreover, Depo-Provera users showed significant increase (p-value-0.02) in BMI compared to their respective pretreatment value (Table 1). The mean change in BMI among Depo-Provera users was 0.67 (+ 3.13%). The maximum increment of BMI among Depo-Provera users was 5.9 kg/m^2^. However, student’s paired t-test showed significant changes in mean weight and BMI among Depo-Provera users, the one-way ANOVA did not show significant (p > 0.05) changes in mean weight and BMI between groups of Depo-Provera users in relation to duration of use (6–24, 27–48 and 51–96 months) (Table 2).

**Table 1:** Mean weight, BMI and MAP of Depo-Provera users

Variables (n = 50)	Mean ± (SD)	Mean differences	Percentage of change	p-value
Weight (Kg)				
Weight after	55.14 ± 6.63	1.6 kg	^+^2.99%	0.02^*^
Weight before	53.54 ± 6.42			
Body mass index (Kg/m^2^)				
BMI after	22.05 ± 3.10	0.67 kg/m^2^	^+^3.13%	0.02^*^
BMI before	21.38 ± 2.70			
Mean arterial pressure (mmHg)				
MAP after	83.21 ± 8.68	0.51 mmHg	^+^0.62%	0.67
MAP before	82.70 ± 7.56			

^+^Increased from baseline; *statistically significant, p-values (obtained by paired t-test)

**Table 2:** Changes in mean weight and BMI of Depo-Provera users related to the duration of use

Parameters	Duration (month)	n	Mean ± SD	Percentage of change	p-value
Change in mean weight (Kg)	6–24	23	1.78 ± 5.06	^+^3.32	0.96
	27–48	21	1.38 ± 4.35	^+^2.58	
	51–96	6	1.66 ± 5.24	^+^3.10	
	Total	50	1.60 ± 4.70	^+^2.99	
Change in mean BMI (Kg/m^2)^	6–24	23	0.76 ± 2.12	^+^3.55	0.95
	27–48	21	0.57 ± 1.78	^+^2.67	
	51–96	6	0.65 ± 2.15	^+^3.04	
	Total	50	0.67 ± 1.94	^+^3.13	

^+^Increased from baseline, p-values (obtained by one-way ANOVA)

#### Magnitude and determinants of change in mean arterial pressure among study participants

Table 3 revealed a non-significant difference (p = 0.85) in the MAP between Depo-Provera users and control group. There was no significant difference in systolic blood pressure (SBP) and diastolic blood pressure (DBP) between Depo-Provera user (108.4 ± 12.47; 70.7 ± 7.82) and controls (108.2 ± 11.00; 72.8 ± 7.83). Furthermore, the change in MAP of Depo-Provera users and control group was not statistically significant (p = 0.67). The mean SBP and DBP were 107 ± 8.86; 70.6 ± 8.18 before and 108.4 ± 12.47; 70.7 ± 7.82 after they had been using Depo-Provera. This difference was not significant (p = 0.41 and p = 0.94, respectively). The present study found no association between the variables, weight (BMI) and blood pressure among the Depo-Provera users (Table 1).

**Table 3:** Comparison of Depo-Provera user and non-user participants using independent t-test

Variables	Depo-Provera users (n = 50)	%	Control group (n = 50)	%	p-value
Age (years)					
< 20	1	2	1	2	0.93
20–30	39	78	40	80	
> 30	10	20	9	18	
Total	50	100	50	100	
Duration of use of Depo-Provera (month)					
6–24	23	46	–	–	
27–48	21	42	–	–	
51–96	6	12	–	–	
Total	50	100	–	–	
Weight (Kg)	55.14 ± 6.62	–	55.52 ± 8.30	–	0.80
Height (m)	1.58 ± 0.65	–	1.60 ± 0.60	–	0.09
BMI (Kg/m^2^)	22.05 ± 3.10	–	21.56 ± 3.05	–	0.42
MAP (mmHg)	83.22 ± 8.68	–	82.91 ± 6.98	–	0.85

Variables statistically significant at p ≤ 0.05, p-values (obtained by independent t-test)

### Discussion

The finding of this study demonstrated that Depo-Provera users had significant weight gain and increased BMI as compared to their respective pretreatment value. This is in agreement with a prospective cohort study performed on 97 Brazilian women, aimed to compare body weight and body composition in Depo-Provera and copper IUD users at baseline and after 1 year of use [11]. Another study, done to assess the association between progestin-only contraceptive use and changes in body weight, revealed that weight gain was greater in Depo-Provera group than in the group using a non-hormonal IUCD, which is in agreement with the findings of the present study [12]. Another similar study showed that the use of Depo-Provera was associated with weight gain compared to the copper IUCD [9]. They also suggested that only the black race was associated with significant weight gain. The present study also supports the findings of other studies where the BMI of the Depo-Provera users was increased significantly than the control group and were in the overweight range [13]. Moreover, a recent study conducted to assess dietary intake and weight gain among adolescents on Depo-Provera, demonstrated that mean BMI increased significantly from 23.7 ± 5.3 at the baseline to 25.3 ± 5.7 after 12 months of using of Depo-Provera with increased mean percentage body fat significantly [14]. While other studies indicated Depo-Provera users pose increased body weight in comparison to their controls, however, changes were not statistically significant [6]. This could be due to the difference in race/ethnicity, which is associated with weight gain, where black women had a greater mean weight gain compared to white, Bushmen, oriental and Papuan with continued Depo-Provera use [9, 16]. It can also be due to practicing physical exercise following good counseling of study participants in the previous study.

Increment of weight is a common phenomenon for women initiating hormonal contraceptives, especially Depo-Provera. However, the existing literature does not provide a clear-cut picture of the mechanism of Depo-Provera-related weight gain. The previous authors tried to report the reasons why the use Depo-Provera can lead to weight increase. Self-reported increase of appetite after 6 months of Depo-Provera use was investigated by Le et al. [17]. This supports Leiman who reported that the weight gain among Depo-Provera users was related to their higher appetite and subsequently higher dietary ingestion as a result of modifications of the hypothalamic appetite control center by Depo-Provera. Contrary to the prior studies, the findings of Lange suggested that Depo-Provera associated weight gain cannot be explained by a simple, direct relationship to the increased food consumption [14, 18]. Therefore, the role of appetite and dietary intake for Depo-Provera-associated weight gain remains to be clarified.

The result obtained from the present study indicates that the Depo-Provera does not have unfavorable effect on blood pressure. The MAP, SBP and DBP between Depo-Provera users and the control group were not significantly different. Changes in MAP, SBP and DBP before and after they had been using Depo-Provera were not statistically significant. This finding was similar with the findings of other studies [6, 15]. This study needs to be extended on wider population so as to generalize the effects of Depo-Provera on the health of Ethiopian women and further studies required to elucidate the molecular mechanism, if any which contribute to the change in body weight among women using Depo-Provera.

## Conclusion

In conclusion, Depo-Provera users had significant weight gain and significant increase in BMI compared with their respective pretreatment value, although, these effects appeared to be independent of the duration of use of Depo-Provera. Women taking Depo-Provera did not show significant change in MAP compared to controls or to their respective pretreatment value, which indicates that Depo-Provera use does not have unfavorable effects on blood pressure.

## Limitation

The possible limitation of this study is the use of small sample size. The other limitation lies in the fact that potential confounders like total daily caloric intake, physical activity and intake of cholesterol rich diets were not considered. In spite of the above limitation, this study tries to assess the possible association between Depo-Provera use and possible weight gain, which is a risk factor for different chronic diseases. So it gives insight for policy makers in considering this effect and to look for other possible solutions.

## Data Availability

The data could be accessed by a request to the corresponding author.

## Abbreviations

MAP: mean arterial pressure
SBP: systolic blood pressure
DBP: diastolic blood pressure
IUCD: intrauterine contraceptive device
BMI: body mass index
